# Language and language disorders: neuroscience to clinical practice

**DOI:** 10.1136/practneurol-2018-001961

**Published:** 2019-07-26

**Authors:** Michael O'Sullivan, Sonia Brownsett, David Copland

**Affiliations:** 1 UQ Centre for Clinical Research, University of Queensland, Brisbane, Queensland, Australia; 2 Department of Neurology, Royal Brisbane and Women's Hospital, Herston, Queensland, Australia; 3 Institute of Health and Biomedical Innovation, Queensland University of Technology, Brisbane, Queensland, Australia

**Keywords:** aphasia, speech therapy, articulation, rehabilitation

## Abstract

Language disorders are common in neurological practice but their accurate recognition and description can be challenging. In this review, we summarise the major landmarks in the understanding of language disorders and the organisation of language in the brain. We describe approaches to assessing language disorders at the bedside or in the clinic as well as the treatment and rehabilitation of aphasia. Finally, we describe how the field of neuroscience is providing new computational and neuroscientific approaches to study the mechanisms of recovery and rehabilitation of aphasia.

## Introduction

Language is pivotal to everyday life and to human culture. The flexibility and vast range of possible combinations in human language exceeds the scope of any other system for vocal communication between primates.^1^ Disorders of this system are common in everyday neurological practice, typically arising from focal injury to the left hemisphere and also from forms of selective neuronal degeneration.^2 3^ Disorders of language are disabling and cause distress to patients, carers and relatives.^4^ The presence of aphasia also creates difficulties in case history taking, assessment and discussion about treatment options and decisions.

Despite being common, language disorders are not always straightforward to evaluate in the clinic or at the bedside. The presentations are varied and there are known pitfalls, such as the mislabelling of fluent aphasia as ‘confusion’. There are various schemes to classify language disorders, which create overlapping terminology (eg, the expressive/receptive and fluent/dysfluent divisions of aphasia, see glossary in box 1). Different disciplines have different traditions and approaches to the analysis of language disorders, which further reduces consistency of terminology. Finally, the approach to the language system in medical textbooks remains dominated by Wernicke–Lichtheim’s 1874 model of the language system and the notion of canonical aphasia syndromes. This view is outdated and often creates misunderstanding.

Box 1 **Glossary**

**Alexia without agraphia**

This is a syndrome characterised by the inability to read with preserved writing. It was conceptualised as a form of disconnection syndrome with a lesion of left primary visual cortex accompanied by a lesion to the splenium of the corpus callosum, cutting off visual input to the angular gyrus and thereby abolishing reading. Writing is intact because left hemisphere language centres remain intact.

**Aphasia**

The term aphasia, interpreted literally, should mean complete absence of language function. However, this situation is so uncommon in practice that the terms aphasia and dysphasia have come to be used interchangeably. This convention is followed in this article and aphasia has been adopted for consistency. Complete loss of speech output is more likely to be due to anarthria, that is, a motor disorder of articulation not limited to language. In this respect, and in contrast to language, anarthria and dysarthria describe qualitatively different deficits.

**Dyspraxia of speech**

This term describes a difficulty converting a motor speech plan into a motor speech action. The patient is typically aware, online, of their inaccurate motor actions. This leads to the attempts to unsuccessfully self-repair those errors, as they occur, which translates into the frequently observed oral ‘groping’ of the speech muscles. The acute awareness and groping features of this disorder are characteristic and can aid in its differential diagnosis.

**Pragmatic language**

This refers to the social use of language, rather than the language itself. It pertains to the rules that govern our use of language in any given context and social interaction. This includes what, how and why something is said, non-verbal communication skills (such as eye contact, facial expressions, body language and so on) and the appropriateness of interactions in a given situation. Importantly, it includes the skills with which we ‘repair’ breakdowns in communication (such as requesting repetition or reforming a question to clarify interpretation).

**Surface dyslexia**

This is a disorder of reading in which people cannot perceive words as single whole entities. As a result, they cannot retrieve their pronunciation from memory. Patients with surface dyslexia can pronounce words using pronunciation rules and therefore pronounce non-words fluently (‘yatchet’) but find irregular words difficult (‘bough’).

## Historical perspective

Pierre Paul Broca’s first report of the famous patient *Tan* was published in 1861.^5 6^ Over the following 4 years, Broca expanded and refined his analysis of disorders of articulatory speech. In 1863, he reported 10 further cases and in 1865 summarised his conclusions in a paper titled, ‘On the site of the faculty of articulated speech’.^7^ Writing just over a decade later,^8^ David Ferrier made it clear that Broca’s conclusions were, by then, widely accepted:

The cause of this affection was shown by Broca—and his observations have been confirmed by thousands of other cases—to be associated with disease in the region of the posterior extremity of the third left frontal convolution, where it abuts on the fissure of Sylvius.

Carl Wernicke’s famous contribution to the understanding of aphasia came later, in 1874.^9^ Wernicke published his paper, ‘*Der aphasische Symptomencomplex*’ at the age of only 26, 4 years after graduation and after only 3 years of neurology training. The crux of Wernicke’s analysis was that disorders of language could occur with lesions in other parts of the brain, not involving the area described by Broca. He described 10 patients, but the notion of Wernicke’s area arose from just one (although another was added as an addendum later): a 75-year-old woman whose severe comprehension deficit made some people believe she was deaf. At postmortem, the only focal lesion was in the first (superior) temporal gyrus on the left.

**Figure 1:**
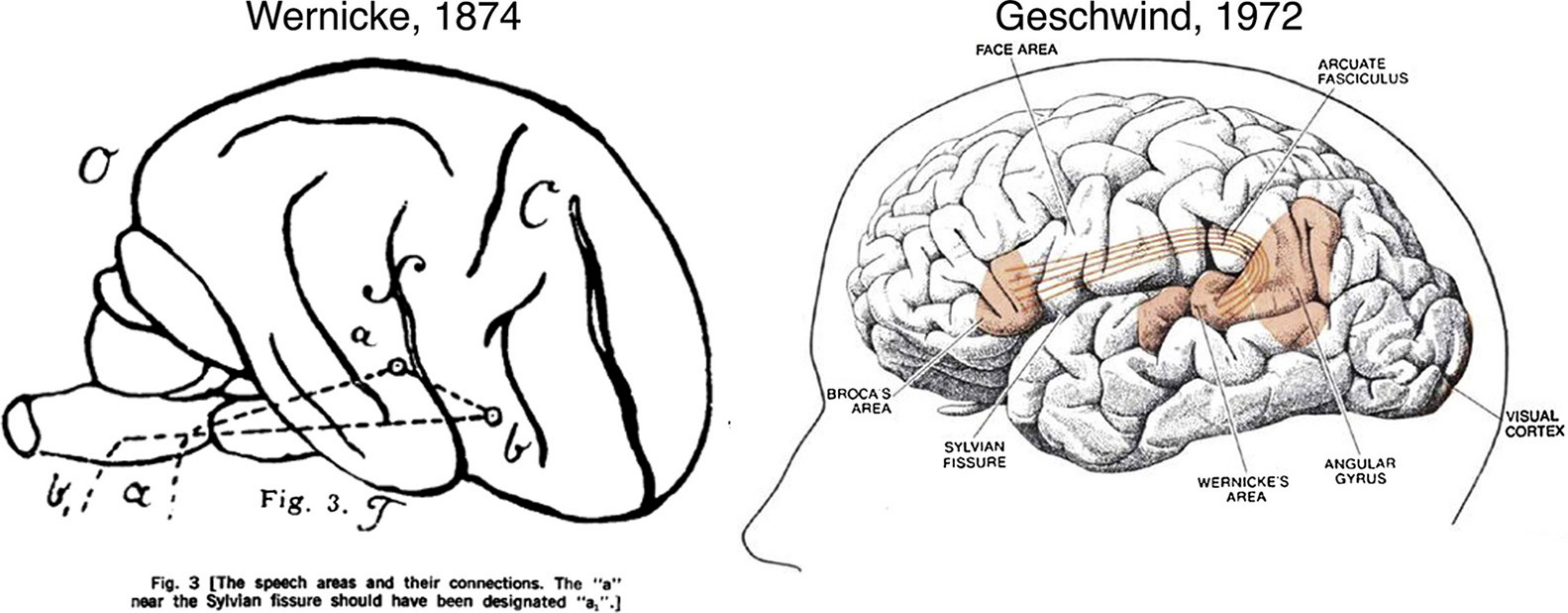
Language models from Wernicke to Geschwind. Wernicke’s original diagram is shown on the left. Geschwind’s contributions included positing that the arcuate fasciculus was the main connection linking Broca’s and Wernicke’s areas, and ascribing a role to the angular gyrus in language (right). Reprinted from Ref. 13: Tremblay P, Dick AS. Broca and Wernicke are dead, or moving past the classic model of language neurobiology. *Brain Lang* 2016;162:60–71, with permission from Elsevier.

Broca’s contributions were made in the French language literature and Wernicke’s in the German, on either side of the 1870–1871 Franco-Prussian war. Nevertheless, Wernicke clearly saw these observations as connected: Wernicke drew a diagram of his proposals for the language network (figure 1)^9^ and also posited that damage to the components of the network he proposed would produce specific patterns of language disturbance. Although Broca and Wernicke now dominate the historical context, they were not the only investigators in the field. Between 1861 and 1874, aphasia was an active and rapidly growing area, attracting the attention of Charlton Bastian and Hughlings Jackson in the UK, among others.^10^

**Figure 2:**
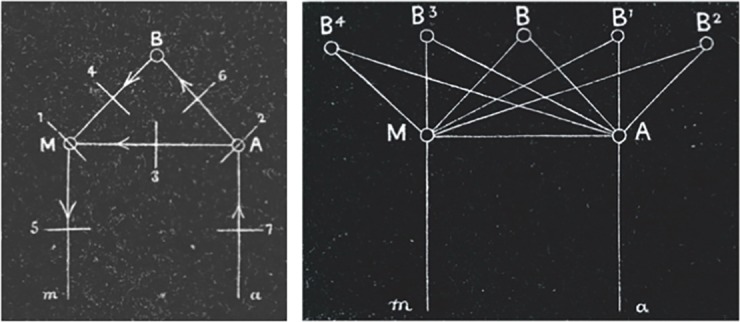
Lichtheim’s language network. ‘M’ represents the motor pole of the network or Broca’s area and ‘A’ the perceptual pole or Wernicke’s area. ‘B’ represents the abstract notion of a ‘concept centre’; another of Lichtheim’s diagrams (right) shows that he was not however arguing that there was a *single* concept centre. The Wernicke–Lichtheim model predicts five patterns of aphasia: (1) Broca’s aphasia; (2) Wernicke’s aphasia; (3) conduction aphasia; (4) transcortical motor aphasia; (6) transcortical sensory aphasia. In addition, (7) could be viewed as depicting ‘pure word deafness’ and (5) the motor speech disorders including apraxia of speech.

In 1885, Lichtheim modified Wernicke’s model of language by adding a ‘concept centre’ (figure 2). This extension accommodates the fact that there are several aspects of normal language function in which repetition plays no role, but which do depend on other mental processes, for example, in producing a monologue based on internal reflections or goals, or silent listening and comprehension. The Wernicke–Geschwind model of the 1960s^11^ additionally included a role for the angular gyrus in silent reading (with input to Wernicke’s area) and Heschl’s gyrus (primary auditory cortex) in silent listening (figure 1).

## Features of aphasia syndromes

Language includes the processes by which thoughts are translated into an ordered pattern of motor output producing speech. At the sensory pole, language processes decode symbols that we see, and sequences of sound that we hear, and link them to representations of words. The observable features of language therefore include syntax, the grammatical structure of sentences, the morphology of words—that is, how speech sounds (phonemes) are combined together—and comprehension, based on both the structure of language and a mental lexicon for words. Aphasia is “…a breakdown in the two-way translation process that establishes a correspondence between thoughts and language.” (Mesulam, 2000).^12^ It follows that aphasia has multiple manifestations, affects the features of linguistic processing that we are able to observe during communication and essentially is part of a two-way breakdown in function.

The features of aphasia depend on the underlying anatomical pattern of pathology. In ischaemic stroke, the most common cause, the clustering of features into aphasia syndromes is mostly a function of underlying vascular anatomy. The superior division of the left middle cerebral artery supplies the inferior and lateral part of the frontal lobe (including Broca’s area), with infarction typically causing dysfluency, agrammatism and disruption of motor aspects of language. Impaired grammatical structure includes greater difficulty with verbs than nouns, and absence of small, linking words (such as prepositions). Even in patients with markedly reduced speech output and dysfluency, automatic ‘overlearned’ phrases are often preserved and can be spoken fluently (eg, ‘the thing is’, ‘know what I mean’ or exclamations—‘for God’s sake!’).

The inferior division of the left middle cerebral artery supplies the lateral temporal lobe, including the region of the superior temporal gyrus nominated as Wernicke’s area. Injury to left lateral temporal regions typically produces fluent speech lacking in meaningful content. The content of speech may be dominated by jargon or clichés and may include neologisms (invented non-words). Patients with posterior lesions are often unaware of the full scale of their language deficit and present with poor self-monitoring skills. They can often detect questions through intonation and respond with fluent answers that are meaningless but sound like language in their melody or intonation. They might appear puzzled that the clinician does not understand them. This pattern of impaired perception of deficit (anosognosia) and poor self-monitoring skills is sometimes a barrier to effective therapy and rehabilitation, both of language and of other impairments.

Disruption to the formulation and structure of language is not limited to individuals with a lesion in the left inferior frontal gyrus and can develop in patients with more posterior lesions. Similarly, comprehension deficits occur in many types of aphasia; careful assessment of an individual with marked difficulty with motor aspects of language will often identify difficulties with comprehension. For example, sentences that require application of syntactic rules for comprehension (eg, *The boy that the girl is chasing is short*) will often reveal impaired comprehension in individuals with deficits in the corresponding motor aspects of language. For these reasons, the division of aphasia into ‘expressive’ and ‘receptive’ types is misconceived and creates a risk of ignoring important deficits, especially in comprehension.

### Repetition

Based on his diagram of the language system, Wernicke reasoned that a lesion affecting the connections alone would produce a distinct type of language disturbance, which he described as *Leitungsaphasie* (which translates as ‘conduction aphasia’). Selective impairment of repetition has been reported in practice and is often accompanied by additional linguistic features that are less easy to understand based on the ‘conduction aphasia’ model. For example, the repetition deficit is greatest for small, grammatical words such as *the*, *if* and *is* (a fact reflected in the Mini-Mental State Examination test of repetition, ‘no ifs, ands or buts’).^13^ Another feature is *conduit d’approche* in which a patient iteratively gets closer to the required phrase with each repetition.

Selective sparing of repetition has been proposed as the hallmark of transcortical aphasia. Transcortical motor aphasia refers to a syndrome of dysfluency and agrammatism in spontaneous conversational speech with relative sparing of repetition. Similarly, the label transcortical sensory aphasia refers to impaired comprehension with relative sparing of repetition (without full access to meaning). The lesions involved in transcortical motor aphasia are often anterior or superior to Broca’s area, though in many cases part of Broca’s area will be involved.^14^ In transcortical sensory aphasia, lesions are typically found in the vicinity of Wernicke’s area.^15^ While there is some controversy about the syndromes, sparing of repetition is an important feature to recognise because it may provide an avenue to exploit in speech and language therapy.

An interesting and extreme example of sparing of repetition was described by Geschwind in 1968, in a case he described as ‘isolation of the speech area’.^16^ The pattern of injury was extensive and multifocal but appeared to spare Broca’s and Wernicke’s areas and the arcuate fasciculus. In this patient, repetition was not only intact but it dominated speech, with marked echolalia:

Despite the notable lack of spontaneous speech, it was noted that she generally repeated questions in a normal voice without dysarthria. Occasionally she would, instead of repeating a phrase, complete it in a conventional manner. Thus to, ‘Ask me no questions’ she would at times reply, ‘Tell me no lies.’… An even more striking phenomenon was observed early in the patient’s illness. The patient would sing along with songs or musical commercials coming over the radio in her room or would recite prayers along with the priest during religious broadcasts.

### Degenerative aphasia syndromes

A syndrome of slowly progressive aphasia, without other features of more generalised dementia, was described in a series of 10 cases by Mesulam in 1982.^2^ This clinical entity came to be known as primary progressive aphasia and subsequent neuropsychological and clinical investigation has led to an increasingly detailed taxonomy and an understanding of the main pathological correlates of these syndromes. There are three broad phenotypes of primary progressive aphasia: progressive non-fluent aphasia, logopenic progressive aphasia and semantic dementia (for a recent and practical review for non-specialists, see Marshall *et al* 17). Monogenic forms of degenerative aphasia may display specific linguistic features, as has been described in people with *Progranulin* mutations.^18 19^

## Clinical–anatomical correlation and networks for language

Models of language such as the Wernicke–Lichtheim model are modular, and therefore predict a certain finite set of typical aphasia syndromes. However, in practice most patients do not fit neatly into a particular recognised subtype, even accounting for our natural bias to fit observations to known syndromes through ‘temptations to see what is not there, to miss what is there and to ignore individual differences’. (McNeil, 1982; p. 698).^20^ Clustering of features is partly driven by vascular anatomy^21^ but this also shows individual variability. Another problem is that simple, constrained models do not account for the role of ‘non-traditional’ regions, including subcortical regions and the right hemisphere, in language disturbance.^22 23^ Since the early days of positron-emission tomography imaging during reading, repetition and articulation,^24^ functional imaging studies have led to two broad shifts in our views of language systems. First, the view has shifted towards concerted activity of multiple regions in a distributed network rather than a more modular viewpoint. Second, functional imaging studies have highlighted the frequent involvement of ‘non classical’ regions, including the right hemisphere, during many language tasks.

Traditional models also have an inconsistent record in terms of predictions about clinical–anatomical correlation. Geschwind proposed that the arcuate fasciculus provided the direct connections between Wernicke’s and Broca’s areas, responsible for verbal repetition.^11^ However, diffusion MRI studies have not found a straightforward relationship between injury or microstructure of the arcuate fasciculus and repetition. Some studies have found an association between arcuate structure and repetition^25^ but others have reported retained repetition, or good recovery of repetition,^26^ in patients with apparent complete interruption of the arcuate connections.^27^ Furthermore, the effects of arcuate injury depend on the site of damage with more anterior lesions producing a more motor pattern of dysfunction.^28^ The notion of the arcuate fasciculus as a simple conduit is therefore incorrect. Furthermore, non-arcuate pathways, such as the uncinate fasciculus and extreme capsule, may also play a role in language function. Some analyses have indicated that more complex models of interaction between regions, such as a dual pathway model, better account for clinico-anatomical correlations.^29 30^ The syndrome of conduction aphasia may also occur with lesions restricted to the cortex, most notably of the supramarginal gyrus.^31^

The cognitive neuropsychological approach differs from traditional models in that it seeks to understand language through component *processes* rather than anatomical modules. The cognitive neuropsychology approach developed from Marshall and Newcombe’s seminal work on dyslexia.^32 33^ Cognitive neuropsychological models offer greater flexibility to explain the huge behavioural variability seen in individual patients. This approach is also more aligned with therapy, where a process can be targeted by specific types of practice routines. This is the dominant framework used by speech and language therapists in the UK and is employed to varying degrees in other countries.

## Assessment of language: the physician

### Conversational and spontaneous speech

Consultations often start with an introduction and an open question. Initial comprehension and then the basic structure, fluency and content of speech are manifest in the answer and conversation that follows. Pauses, lack of linking words, disrupted grammatical structure and paraphasic errors are some of the features that might be noted.

### Naming

A common approach on a ward round is to ask patients to name nearby objects, typically starting with items such as ‘pen’, ‘watch’, ‘cup’ or ‘jug’, but this approach has limitations. Naming becomes more difficult in moving from high frequency to low frequency objects and words. An ideal assessment would be graded, giving some idea of the severity of naming difficulty. However, a graded approach is difficult with opportunistically identified objects. There is often a leap in difficulty once the high frequency items such as ‘pen’, ‘watch’ or ‘jug’ have been used, to other items that may be at the bedside but are rarely encountered outside a hospital (eg, ‘stethoscope’, ‘drip stand’). The naming section of the Addenbrooke’s Cognitive Examination^34^ provides 12 objects of graded difficulty (ranging from ‘pen’ and ‘watch’ to ‘accordion’) and the Queen Square Book also provides 10 more challenging objects for naming (such as ‘lobster’ and ‘owl’). Using one of these resources, in a few additional minutes, adds sensitivity and a degree of quantification.

### Comprehension

Testing comprehension at the bedside most commonly employs staged commands beginning with simpler single–stage commands (‘lift up your hands’, ‘close your eyes’). It is important to remember that, in general, comprehension is tested through the accuracy of a subsequent motor response, so other types of deficit can interfere beyond initial comprehension. For example, lateralised commands (‘touch your left ear’) can be affected by left–right disorientation. At a very simple level, a patient with a new hemiplegia may become distressed by their difficulty with the required motor component and ignore any nuances of the language component. It is also important to distinguish errors due to disorders of spatial processing or limb dyspraxia from poor initial comprehension.

### Repetition

The phrase from the Mini-Mental State Examination, ‘No ifs, ands or buts’ is used frequently. The original intention was probably to define a phrase devoid of real meaning to provide a relatively pure measure of repetition (not influenced by access to semantic information). However, the phrase does not achieve this fully as it still contains real rather than non-words. ‘If’, ‘and’ and ‘but’ are all linking words, and the presence of linking words makes this phrase sensitive to conduction aphasia, for reasons that are not fully clear. One logical approach to testing repetition is to start with single words and increase the difficulty from simple monosyllabic words (‘cat’) to words of increasing length and complexity. The Addenbrooke’s Cognitive Examination provides some examples of especially difficult single words that can increase the sensitivity to subtle deficits (eg, ‘unintelligible’).

### Reading and writing

Reading and writing should be included in a thorough examination of language. Assessment will often identify deficits consistent with spoken language. For example, impaired comprehension of words presented visually and impaired grammatical structure of written language may correlate with deficits in conversational language. Assessment of reading and writing can also uncover disorders that do not affect spoken language such as *alexia without agraphia* and *surface dyslexia* (see glossary in box 1).

## Assessment: speech and language therapist

Speech and language therapy assessment is linked from the outset to both identifying and accurately characterising impairment and defining possible approaches to therapy. It is less concerned with aphasia classification, which currently has no role in defining therapy. One early priority is to establish the amount of support a patient requires while on a ward in order to address their needs. Is their understanding aided by written words? Do short sentences need to be used? Do they need pictorial support? Can they reliably understand the questions posed to them?

Initial bedside assessment might include conversational speech and confrontation naming of objects, with difficulties triggering a more detailed assessment of naming skills. Basic bedside assessment of spoken comprehension skills might typically include a range of instructions designed to tap into the patient’s ability to follow increasingly complex commands, focusing on the number and classes of key words that are understood. Testing of comprehension is usually explicitly linked to purposeful function. For example, the speech and language therapist might introduce three items—a cup, a spoon and a fork—and then give related instructions: ‘Here is a cup, a fork and a spoon. Put the fork in the cup’. This approach to comprehension provides information relevant to activities of daily living and can provide useful information to other therapy disciplines.

Several repetition skills are usually tested to investigate different possible levels of breakdown. For example, comparing non-word repetition with real word repetition investigates the patient’s reliance on the semantic system in order to repeat an item. Thus, in theory, the word ‘cat’ can be repeated via an ‘indirect route’ from auditory regions to articulatory regions via the semantic system, or directly via a ‘direct’ sound-to-sound correspondence without accessing the semantic representation of an item. However, the non-word ‘blorf’ cannot be repeated via the indirect route, as the patient has no semantic representation of this. Failure to repeat a real word correctly does not necessarily indicate a repetition deficit per se but could indicate a semantic access deficit.

Individualised assessment includes carefully defining the level of residual function if significant problems are identified. If a patient’s assessment indicates significant difficulty with comprehension, then the speech and language therapist might ‘step-down’ the assessment to establish if the patient has a reliable ‘yes/no’ response. Thus, if they have responded ‘yes’ to a question, have they: (1) understood the question and (2) is ‘yes’ their intended response or did they say yes merely by chance? In the acute setting, therapists are frequently concerned by the assumption that a patient’s ‘yes/no’ response is reliable.

### Reading and writing

In the acute setting, assessment of language in the written domain forms an essential component of both formal and informal screens. Often the primary aim of assessment is to ascertain if there is damage to this modality or to establish the use of this modality as a means of supporting communication. Patients may be unable to follow complex instructions in the spoken domain but be more successful if the same instructions are written down. Likewise, a person with certain types of articulatory speech difficulty, especially apraxia of speech and dysarthria, may have to rely on writing to support their expressive language to allow them to communicate effectively with others.

### Pragmatic language

Pragmatic deficits can arise with lesions not expected to affect language, such as in the right hemisphere, and create a significant—often unacknowledged—impairment in a patient’s overall ability to communicate. For example, apparently rude or abrupt behaviour might arise when a patient cannot control the tone or volume of their voice, or has a new inability to perceive, or rely on, facial expressions to support communication. Speech and language therapists routinely assess pragmatic language and can provide valuable information to aid communication with patients and carers.

### Formal language assessments

Quantified assessment batteries can often supplement informal assessment in an inpatient setting. For conversational speech, quantified assessment can be based on a more detailed sample of a person’s language production skills (often around 2–3 min). This can be done though tasks such as describing a complex picture or through story retelling. There are several available standardised assessments of naming skills. Selecting an appropriate test is based on several factors including the severity of the suspected impairment, with tests such as the Boston Naming Test being most appropriate for the more severely impaired, and the Graded Naming Test (or the Philadelphia Naming Test in the USA) for those likely to have more subtle naming deficits.

## Treatment and rehabilitation of aphasia

There are several proposed approaches for speech and language therapy interventions to treat aphasia. These include: the didactic (reteaching language); behavioural modification (reteaching language using principles from behavioural psychology); the stimulation school (re-accessing intact language by providing ample stimulation); pragmatics (optimal use of unimpaired skills to promote communication by any strategy possible) and cognitive neuropsychology (interventions based on theories of language processes and their disruption). In contrast to the ‘deficit-reducing’ or ‘impairment-based’ approaches to therapy, the pragmatic or ‘functional’ approach emphasises functional communication rather than recovery of language skills (compensation rather than restitution). However, advocates of a more recent approach to therapy, known as constraint-induced aphasia therapy, argue that pragmatic schools of therapy actually increase the linguistic impairment through its inherent non-usage of linguistic skills. Constraint-induced aphasia therapy is based on principles that experience (or ‘use’) enhances a system, whereas lack of experience (or ‘non-use’) can cause it to atrophy (see Pulvermuller *et al*, 2001).^35^

In current practice, we use a mix of approaches. Indeed, the Royal College of Speech and Language Therapists clinical guidelines advocate a multilayered approach that ‘minimises the disability, addresses emotional health, and enables participation’. Typically, reducing ‘the disability’ might be achieved through a combination of identifying and targeting the precise level of breakdown while simultaneously supporting alternative communication methods. ‘Enabling participation’ might typically involve both educating family members about the impact of aphasia and the techniques that can be employed to support communication outside the clinical setting.

The most recent Cochrane review of aphasia therapy concluded that: ‘Our review provides evidence of the effectiveness of speech and language therapy for people with aphasia following stroke in terms of improved functional communication, reading, writing, and expressive language compared with no therapy’.^36^ There is a lack of evidence on the optimal approach for the delivery of speech and language therapy, which recent trials are beginning to address.^37^ Other major questions to address include how much treatment an individual requires, and the optimal intensity or scheduling of therapy (massed vs distributed practice).^38^ There are recognised challenges to methodologically sound randomised-controlled trials of aphasia therapy, which include difficulty in blinding, identifying suitable control interventions and ensuring standardisation of therapy.^39^

There have been several studies of pharmacological interventions to improve language outcome after stroke, with some evidence for the effectiveness of memantine and piracetam. However, questions about piracetam’s safety persist.^40^ There is also emerging evidence for the role of selective serotonin reuptake inhibitors in aphasia recovery depending on lesion site.^41^ To date, there is no convincing evidence for using stimulation strategies such as transcranial magnetic or direct current stimulation,^42^ although recent studies have shown promising results when aphasia therapy is paired with stimulation of the motor cortex applied to preserved left temporal lobe regions.^43^ A promising strategy, moving forward, will be to test combined interventions of pharmacological, neurophysiological and behavioural approaches.

## Neuroscience of language recovery: prospects for new approaches

**Figure 3:**
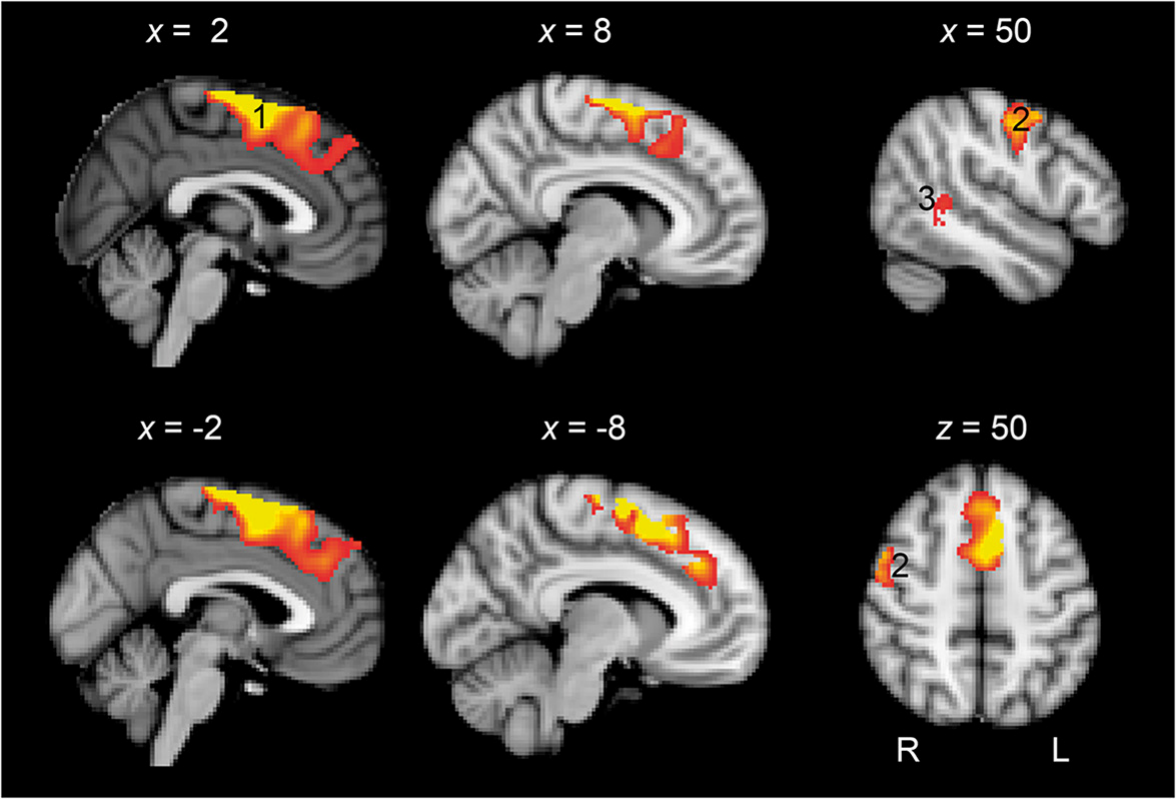
Domain-general cortex and language recovery. Standard T1-weighted anatomical slices overlaid with functional MRI activity correlating with language recovery. A large cluster of activity was observed in the preSMA extending to dorsal mid-cingulate cortex and dACC (1). Activity was also observed in the right precentral and postcentral gyri (2) and right posterior superior temporal gyrus. (Rendered at p<0.05 with 200 voxel extent. Peak voxel whole brain significant at p<0.001 with family-wise error (FWE) correction for multiple comparisons.). dACC, dorsal anterior cingulate cortex. Reprinted from Geranmayeh *et al*. Domain-general subregions of the medial prefrontal cortex contribute to recovery of language after stroke. *Brain* 2017;140:1947–58.

There has been recent interest in the role of domain-general networks (ie, networks in the brain that are not specialised for language or any other single cognitive domain) in the recovery of language. The coexistence of damage to networks involved in attention and executive control may worsen communication deficits acutely^44^ and limit the potential for improvement over time.^45 46^ Some regions labelled as domain-general (or alternatively as multiple-demand cortex) include the dorsal anterior cingulate cortex (figure 3) and anterior insula. These regions are engaged in the allocation of cognitive resources to challenging tasks.^47^ The frequent involvement of these regions in language tasks in the recovery phase of aphasia has led to the suggestion that external stimulation of these regions might hasten recovery. In support of these idea, transcranial magnetic stimulation targeted at multiple-demand cortex in the medial frontal lobe enhanced learning of a novel vocabulary in a study of healthy participants.^48^

The heterogeneity of aphasia—and especially the difficulty of predicting outcome in any individual patient—also hinders trials. Imaging and computational approaches are being applied to tackle this problem. The PLORAS (Predicting Language Outcome and Recovery After Stroke) database brings together neuroimaging and outcome data on over 800 people with stroke,^49^ leading to the prospect of accurate outcome prediction based on the anatomy of initial injury.^50^ Potentially, advanced MRI can also delineate variations in anatomy of undamaged brain regions that influence the capacity for recovery after injury.^51^ Translation of this type of approach into practice has the potential to lead to personalised medicine approaches to therapy and more efficient clinical trials.

**Key Points**

- Language deficits are common, disabling and distressing for relatives and carers.
- The Wernicke–Lichtheim model is outdated and individual deficits often do not fit classical syndrome descriptions.
- Clinicians should assess the elements of language function and be descriptive; this approach also helps in planning individual therapy.
- Speech and language therapy following stroke improves functional communication, reading, writing and expressive language, though with many unanswered questions about its timing, quantity and optimal approach.
- Advances in understanding brain networks and processes involved in functional recovery is leading to novel therapeutic approaches, with possible implications for treating cognitive disorders other than aphasia.
